# Podcasting in Medicine: The Current Content by Emergency Medicine Subspecialty

**DOI:** 10.7759/cureus.9848

**Published:** 2020-08-18

**Authors:** Andrew Little, Andrew Kalnow, Amber R Walker, Patricia Capone

**Affiliations:** 1 Emergency Medicine, AdventHealth East Orlando, Orlando, USA; 2 Emergency Medicine, OhioHealth Doctors Hospital, Columbus, USA; 3 Emergency Medicine, Ohio University Heritage College of Osteopathic Medicine, Athens, USA; 4 Emergency Medicine, Ohio University Heritage College of Osteopathic Medicine, Dublin, USA

**Keywords:** podcast, emergency medical service, toxicology and envenomation, simulation medicine, ultrasound (u/s), wilderness medicine

## Abstract

Background

Podcasts and their use in medical education, particularly emergency medicine (EM), are growing and becoming more popular. Many podcasts focus on EM, but the number of podcasts on each EM subspecialty remains unknown. Therefore, the goal of this study was to ascertain the number of podcasts available by EM subspecialty and collect the basic characteristics of each podcast.

Methods

We conducted a Google-based, investigational study of EM podcasts by subspecialty from July 2019 to January 2020. Search terms included “podcasts in ____”, where the EM subspecialties of Toxicology, Ultrasound, Wilderness Medicine, Emergency Medicine Services, Medical Education, and Simulation were inserted to identify podcasts.

Results

Emergency Medical Services (EMS) and Medical Education subspecialties have the most active podcasts. Toxicology and EMS have the most inactive podcasts, while Medical Education and Simulation were the only subspecialties found to not have any identified inactive podcasts.

Conclusions

The use of podcasts in EM has been increasing overall, but physicians in specific subspecialties, such as EMS and Medical Education, have access to a larger number of podcasts specific to their subspecialty than others. There is an opportunity for experts in Toxicology, Simulation, and Ultrasound to create podcast content.

## Introduction

Over the past two decades, the availability and utilization of medical podcasts have grown substantially, specifically within the specialty of emergency medicine (EM) [1]. With this growth, podcasts have become a major source of knowledge dissemination within medicine [2,3]. A 2018 trade report estimated that 124 million people listen to podcasts, approximately 73 million had listened within the last month, and the average listener consumed seven podcasts per week [4]. Looking at just the Apple Podcast platform, there are more than 500,000 active podcasts in 100 languages, exemplifying the diverse reach of podcasting for information and entertainment dissemination [5,6].

The utilization of podcasts in medicine has seen a similar growth and depth to that of podcasting in general, with specific podcasts targeting almost every discipline and specialty within medicine [2,7]. This study represents a Google-based assessment of currently free podcasts by specific subspecialties in EM, with the specific aim of better defining the availability, content, and characteristics surrounding these podcasts. While looking broadly at specialty-specific medical podcasts, we did not differentiate content areas within each specialty.

## Materials and methods

We conducted a Google-based, investigational study of free EM podcasts by subspecialty from July 2019 to January 2020. Search terms included “podcasts in ____”, where the EM subspecialties of Toxicology, Ultrasound, Wilderness Medicine, Emergency Medicine Services, Medical Education, and Simulation were inserted to identify current podcasts covering these topics. The authors felt that these were the major subspecialties germane to EM that did not have significant overlap with other specialties, such as Pediatric Emergency Medicine being both an EM and Pediatric subspecialty. We searched within the top 50 results, consistent with previous studies performed. Once a podcast was identified to meet our criteria, we collected the following information: name, active status, number of episodes, frequency of release, average episode length, and podcast website. We deemed active status as a podcast that had released content in the previous six months and attempted to determine if episodes were released on a regular schedule based on frequency.

## Results

Overall, we found 40 podcasts covering all of the search specialties, with EMS as the subspecialty with the most podcasts and Simulation with the fewest. Medical Education had the most podcast episodes available. Individual subspecialty results are discussed below.

Toxicology

For Toxicology, there were a total of eight podcasts found, five of which were active. There were a total of 208 episodes, with 46% of the content coming from two podcasts. After removing these two podcasts from the data, the remainder of the podcasts averaged 19 episodes per podcast. All Toxicology podcasts provide content sporadically, which was common for the other subspecialties. Toxicology is tied for the third most active podcasts (with Ultrasound) and has the fourth-highest number of episodes. The average podcast episode duration in Toxicology is 39 minutes (Table 1).

**Table 1:** Toxicology podcasts with activity status, the total number of episodes, average episode length, and release schedule

Podcast Name	Active?	Number of Episodes	Release Schedule	Average Length (minute)
Oregon 1-800-222-1222	Yes	51	Sporadic	82
The Journal of Medical Toxicology Podcast	No	28	Sporadic	50
Tox in Ten	Yes	13	Sporadic	11
The Poison Review Podcast	No	15	Sporadic	37
ToxNow	No	45	Sporadic	23
The Dantastic Mr Tox and Howard	Yes	26	Sporadic	67
ToxChats	Yes	25	Sporadic	15
Dose Makes The Poison: The Toxcast	Yes	5	Sporadic	25

Ultrasound

For Ultrasound, we found a total of six podcasts, five of which were active. There were a total of 314 episodes with one podcast providing 38% of the content. After removing this outlier, the remainder of the podcasts averaged 39 episodes per podcast. All the Ultrasound podcasts provide content sporadically, except for one podcast that provides content bimonthly. Ultrasound is tied for the third most active podcasts (with Toxicology) and has the third-highest number of episodes. The average podcast episode duration in Ultrasound is 22 minutes (Table 2).

**Table 2:** Ultrasound podcasts with activity status, the total number of episodes, average episode length, and release schedule

Podcast Name	Active?	Number of Episodes	Release Schedule	Average Length (minute)
Ultrasound Podcast	Yes	120	Sporadic	25
Ultrasound GEL	Yes	84	Sporadic	19
5 Minute Sono	Yes	50	Sporadic	4
Sound Sports Imaging: Ultrasound Podcasts	No	2	Sporadic	5
International Sonography Podcast	Yes	20	Sporadic	55
FOCUS on POCUS	Yes	38	Bimonthly	21

Wilderness medicine

For Wilderness Medicine, there were a total of four podcasts found, three of which were active. There were a total of 51 episodes with one podcast providing 49% of the content. After removing this outlier, the remainder of the podcasts averaged nine episodes per podcast. As far as the frequency of content being published, all of the Wilderness Medicine podcasts provide content sporadically. This subspecialty has the fifth-highest number of active podcasts and the least number of episodes. The average podcast episode duration in Wildness Medicine is 35 minutes (Table 3).

**Table 3:** Wilderness Medicine podcasts with activity status, the total number of episodes, average episode length, and release schedule CWS: The Center for Wilderness Safety, WEM: Wilderness and Environmental Medicine.

Podcast Name	Active?	Number of Episodes	Release Schedule	Average Length (minute)
RAW Medicine	Yes	9	Sporadic	60
WEM Live!	Yes	16	Sporadic	44
The CWS Podcast	No	1	N/A	13
Exploration Medicine	Yes	25	Sporadic	24

Emergency medical services

For Emergency Medicine Services (EMS), we found a total of 12 podcasts, nine of which were active. There were a total of 852 episodes with two podcasts providing 44% of the content. After removing these two outliers, the remainder of the podcasts averaged 48 episodes per podcast. All but two of the EMS podcasts provide content sporadically similar to the Medical Education podcasts. The EMS subspecialty has the highest number of active podcasts and the second-highest number of episodes. The average podcast episode duration in EMS is 30 minutes (Table 4).

**Table 4:** Emergency Medical Services podcasts with activity status, the total number of episodes, average episode length, and release schedule MCHD: Montgomery County Health District, EMS: emergency medical services, PEC: Prehospital Emergency Care, CBCEMP: Columbia and Boone County Emergency Medical Professionals.

Podcast Name	Active?	Number of Episodes	Release Schedule	Average Length (minute)
MedicCast	No	75	Sporadic	25
The FlightBridgeED Podcast	Yes	175	Sporadic	43
MCHD paramedic podcast	Yes	73	Sporadic	22
The Medic Assessment	Yes	25	Sporadic	42
Medic2Medic Podcast	Yes	199	Weekly	33
PEC Podcast	Yes	76	Sporadic	44
EMS in Wisconsin Podcast	Yes	23	Monthly	43
Austin-Travis County EMS System Office of the Medical Director	Yes	32	Sporadic	14
EMS Nation	Yes	91	Sporadic	24
Medic 101 Podcast	No	6	Sporadic	28
Curbside to Bedside	Yes	24	Sporadic	34
CBCEMP Podcast	No	53	Sporadic	13

Medical education

For Medical Education, there were a total of eight podcasts found, all of which were active. We noted a total of 1349 episodes with one podcast providing 40% of the content. After removing this outlier, the remainder of the podcasts averaged 116 episodes per podcast. Similar to the EMS subspecialty, all but two of the Medical Education podcasts provide content sporadically. The Medical Education subspecialty has the second-highest number of active podcasts and the most number of episodes. The average podcast episode duration in Medical Education is 26 minutes (Table 5).

**Table 5:** Medical Education podcasts with activity status, total number of episodes, average episode length, and release schedule EM: emergency medicine.

Podcast Name	Active?	Number of Episodes	Release Schedule	Average Length (minute)
Medical Education Podcasts	Yes	100	Sporadic	16
KeyLIME	Yes	250	Weekly	25
The Medutopia Podcast	Yes	21	Sporadic	35
The Spark: Medical Education Podcast	Yes	14	Sporadic	21
Emergency Medicine Cases	Yes	134	Monthly	43
Emergency Medical Minute	Yes	536	Sporadic	9
EM Basic	Yes	88	Sporadic	22
Academic Life in Emergency Medicine	Yes	206	Sporadic	33

Simulation

For Simulation, we found a total of two podcasts, both of which were active. We found a total of 190 episodes, and each podcast providing an equal proportion of content. The podcasts had an average of 95 episodes per podcast. One podcast provides content sporadically; the other provides content weekly. Simulation has the fewest active podcasts and the fifth-highest number of episodes. The average podcast episode duration in Simulation is 30 minutes (Table 6).

**Table 6:** Simulation podcasts with activity status, total number of episodes, average episode length, and release schedule

Podcast Name	Active?	Number of Episodes	Release Schedule	Average Length (min)
Simulcast	Yes	94	Sporadic	28
The Center for Medical Simulation	Yes	96	Weekly	32

## Discussion

The use of podcasts to educate and inform physicians has been rapidly expanding; most recently graduated physicians and those still in training are increasingly exposed to podcasts during their formal and continuing education [1,8-10]. While it has been shown that medical podcasts, in general, have increased in number and popularity over the past 20 years, a recent study had cataloged current medical podcasts and determined EM had the largest number of podcasts and the most available content [2,7]. This study further expands on the variability of podcast distribution by each searched EM subspecialty. EMS was found to have more active and inactive podcasts, but Medical Education was found to have a much higher amount of content, with 1.5-times the number of available episodes as EMS and 4-6-times the number of episodes as Ultrasound and Toxicology (the next two most active subspecialties). Other EM subspecialties had limited content available, which provides a gateway for members of these subspecialties to publish additional educational opportunities in what many learners have found to be a favorable means of obtaining medical content and potentially continuing medical education credit [1,2,7-10].

Limitations

Our study was limited in that it was based solely on internet searches to obtain podcast information. There may be a number of additional podcasts available that were hidden from our search parameters due to search engine optimization settings or the podcast producers not having an official podcast website (outside of their hosting service). As we chose to only search for podcasts using a Google search and not directly on podcast hosting sites such as Apple Podcast or Stitcher. There could have been other podcasts behind paywalls or hosted only on podcasting platforms that we were unable to evaluate. Opportunities for further research include evaluating learner utilization of the podcasts found, as it is unknown if the number of listeners affects the number of podcasts and the creation of content in each subspecialty.

## Conclusions

While the use of podcasts in EM has been increasing overall, it is more evident in certain subspecialties, particularly EMS and Medical Education. As this study only looked at the content provided by each podcast, we cannot make conclusions about the quality of the episodes or learner utilization. This study aimed to expand on the recent study about medicine specialty podcasts to provide a categorically indexed review of EM subspecialties within the search parameters outlined above. EM learners who use medical podcasts will ideally find this article helpful in identifying recent content in their specific area of interest.
